# Variation in koala microbiomes within and between individuals: effect of body region and captivity status

**DOI:** 10.1038/srep10189

**Published:** 2015-05-11

**Authors:** Niccoló Alfano, Alexandre Courtiol, Hanna Vielgrader, Peter Timms, Alfred L. Roca, Alex D. Greenwood

**Affiliations:** 1 Leibniz Institute for Zoo and Wildlife Research, Berlin, Germany; 2 Tiergarten Schönbrunn, Vienna, Austria; 3 University of the Sunshine Coast, Sippy Downs, Queensland, Australia; 4 Department of Animal Sciences, University of Illinois at Urbana-Champaign, Urbana, Illinois, USA

## Abstract

Metagenomic analysis of 16S ribosomal RNA has been used to profile microbial communities at high resolution, and to examine their association with host diet or diseases. We examined the oral and gut microbiome composition of two captive koalas to determine whether bacterial communities are unusual in this species, given that their diet consists almost exclusively of *Eucalyptus* leaves. Despite a highly specialized diet, koala oral and gut microbiomes were similar in composition to the microbiomes from the same body regions of other mammals. Rectal swabs contained all of the diversity present in faecal samples, along with additional taxa, suggesting that faecal bacterial communities may merely subsample the gut bacterial diversity. Furthermore, the faecal microbiomes of the captive koalas were similar to those reported for wild koalas, suggesting that captivity may not compromise koala microbial health. Since koalas frequently suffer from ocular diseases caused by *Chlamydia* infection, we also examined the eye microbiome composition of two captive koalas, establishing the healthy baseline for this body part. The eye microbial community was very diverse, similar to other mammalian ocular microbiomes but with an unusually high representation of bacteria from the family Phyllobacteriaceae.

The koala, *Phascolarctos cinereus*, is an arboreal marsupial that has a unique diet consisting almost exclusively of *Eucalyptus* sp. leaves. Eucalyptus foliage has been described as an “unpromising” dietary source, low in nutrients and proteins but at the same time rich in oils and secondary plant compounds, such as lignin, cellulose and tannins, which are toxic to most animals^1^,^2^. Koalas have evolved a set of behavioral, physiological, morphological and metabolic adaptations to such a diet^3^. For example, they have a specialized digestive tract with an extremely enlarged caecum^4^ and very long retention times of food within the gut^5^. Koalas can thus break down plant material by fermentation and enzymatic degradation, and finally extract sufficient nutrients to maintain active metabolism. Bacteria are thought to play an important role in this process. Several different microorganisms that are able to degrade lignin and tannins have been isolated from the koala gastrointestinal tract^6^,^7^. However, whether such an exclusive diet influences the composition of koala bacterial communities, or microbiomes is unknown.

Recent developments in culture-independent methods based on large-scale comparative analyses of 16S ribosomal RNA and metagenomics have the potential to profile microbial communities at high resolution even in complex environments like the intestinal microbiota^8^. Such methods have therefore been employed to study how the composition of bacterial communities relates to the diet in several species^9^,^10^,^11^. For certain organisms such as humans and mice^12^,^13^, the relationship between diet and microbiome can be directly studied by modifying the diet of some individuals and assessing how the microbiome is being influenced by such change. This experimental approach has the benefit of effectively isolating the influence that diet exerts upon the microbiome from the influence of many other factors known to impact the microbiome. Unfortunately, this approach cannot be applied to koalas because of their extreme dietary specialization. Instead, fully assessing the extent to which koala’s microbiome is specific to its unique diet requires profiling microbial gut communities in a representative sample of koalas and comparing the profiles to those of other animals.

Because wild koala samples can be difficult to acquire and invasive sampling of captive koalas is discouraged, defining an effective sampling strategy is essential. A recent study^14^ employed high-throughput GS FLX pyrosequencing to describe the composition of the koala microbiome across the hindgut in two wild koalas. This demonstrated that the koala hindgut microbiome is a complex and diverse environment and that the bacterial communities vary considerably in different regions of the intestine. However it is unclear whether the samples are representative of the entire gut, and whether or not widely used noninvasive samples such as faeces would provide an accurate representation of host microbiome. To the best of our knowledge, there have been no comparative studies based on high-throughput sequencing addressing whether rectal swabs and faecal samples yield consistent results in wild mammal gut microbiome research. Therefore, whether faecal samples are a good proxy to profile the gut microbiome in mammals in general, and in koalas in particular, remains to be determined.

The microbiome is known to vary both among individuals and among populations living in different environments. For example, shifts in gut microbiome composition between wild and captive individuals have been highlighted in several mammalian species, such as primates^15^,^16^, goats^17^, red pandas^18^ and giant pandas^8^. The microbiome differences may be a consequence of the artificial nature of the zoo environment, particularly dietary changes. Thus, whether or not captive koalas can be used to study the diet specialization of the microbiome remains to be established.

For this study, rectal swabs and faeces were sampled from two captive koalas from the Tiergarten Schönbrunn in Vienna (Austria): a 14 year old male (SN241) and a 12 year old female (SN265). Although previous studies focused on the gut microbiome, initial digestion takes place in the mouth and thus koalas could be expected to have a unique microbiome in this compartment. Therefore, we also obtained oral swabs from the two koalas. Moreover, we sampled the eye microbiome of our two captive koalas. This body region was included to obtain a comparison point independent to digestion associated organs and to establish a baseline for the microbiome of healthy koala eyes, since wild and captive koalas frequently suffer from ocular infections caused by the highly prevalent *Chlamydia*, which is regarded as the primary disease threat to the species^19^,^20^. Such high incidence of chlamydial infection has been correlated with the presence of the recently described koala retrovirus (KoRV)^21^. All samples were characterized by 16S rRNA high-throughput Illumina sequencing. From a total of 1,956,592 quality-filtered reads, we identified 7,843 operational taxonomic units (OTUs) defined at the 97% similarity level. We first described the bacterial communities from the different body parts and compared our results with microbiome studies on other mammalian species. Second, we compared the gut microbiome profiles obtained by high-throughput sequencing from rectal swabs and faecal samples and discuss the reliability of using faeces as non-invasive sampling method in microbiome studies. Finally, we assessed whether captivity plays a role in shaping the koala faecal microbiome by comparing results from captive animals with existing data on wild koalas.

## Results & Discussion

### General microbiome characteristics

**Figure 1:**
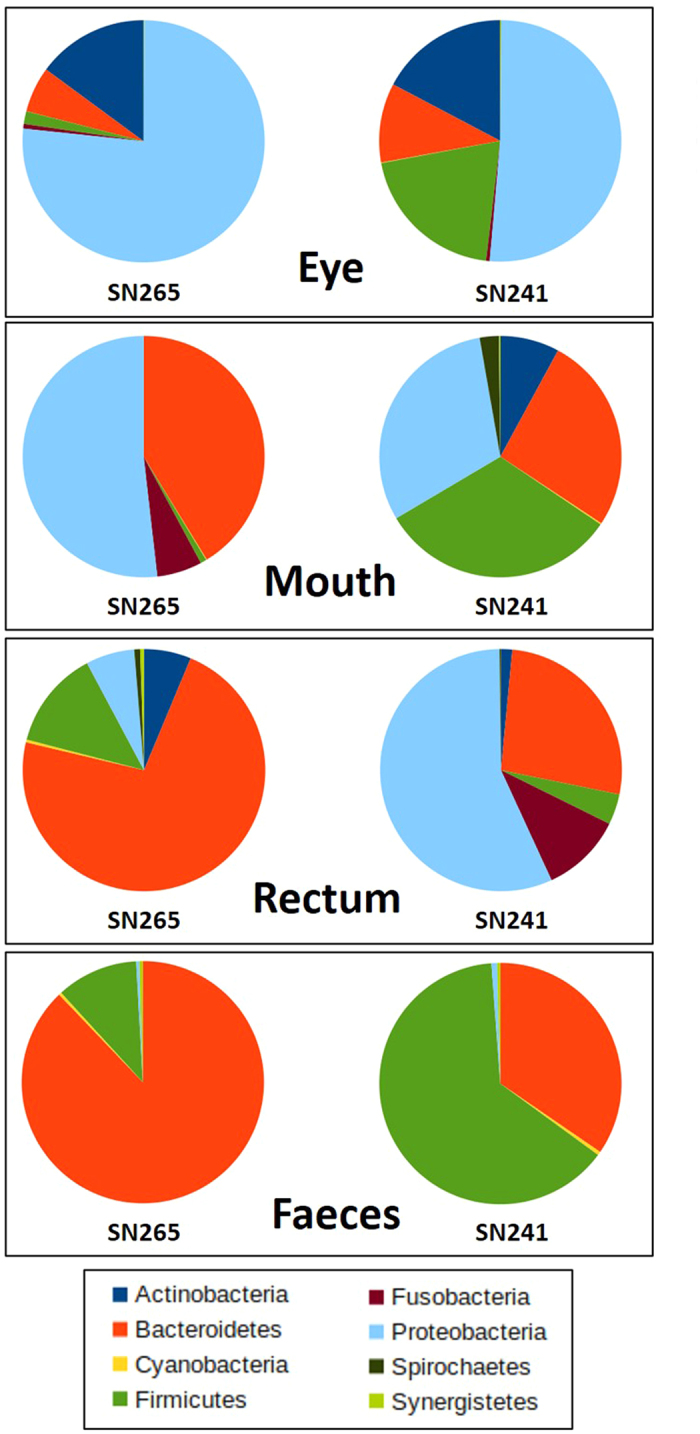
Relative abundance of bacterial phyla. Pie chart representation of the relative abundances of the most common phyla found in the eye, mouth, rectum and faeces of the two koalas. The relative abundance values of each phylum for each sample are reported in Suppl. Tab. 1.

**Figure 2:**
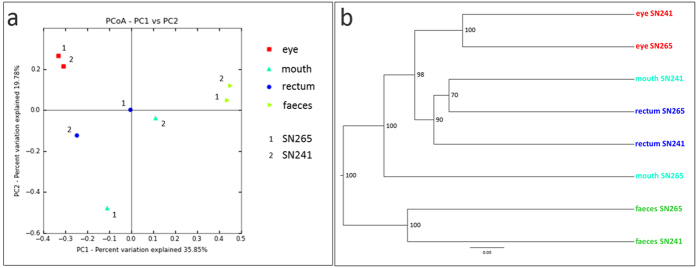
Principal Coordinate Analysis (PCoA) and UPGMA tree of Unifrac distances of the eye, mouth, rectal and faecal samples of two captive koalas. Panel A represents a 2 dimensional unweighted PCoA plot by sample type. Panel B represents the UPGMA clustering on unweighted Unifrac distances. Nodal supports were assessed by jackknife using 1,000 replicates. The two plots highlight that the samples tended to cluster by body region but not by koala specimen.

The composition of microbial communities was similar between samples when analyzed at low taxonomic resolution. Bacteroidetes (6.08–87.64%), Firmicutes (0.81–63.61%) and Proteobacteria (0.40–76.56%) were the most abundant phyla across most of the samples, followed by Actinobacteria and Fusobacteria (Fig. 1; Suppl. Fig. 1; Suppl. Tab. 1). At a higher taxonomic resolution, the different parts of the koala gastrointestinal tract and the koala eye were characterized by distinct bacterial communities (Fig. 2A). Indeed, principal coordinate analysis of unweighted UniFrac distances, which measure the similarity between bacterial communities based on phylogenetic distances, showed that koala microbial communities clustered by body region (Fig. 2A; Suppl. Fig. 2A). No clustering pattern was evident based on the weighted PCoA (Suppl. Fig. 2B), but the unweighted PCoA was consistent with the UPGMA tree (Fig. 2B). Irrespective of the method, no clustering pattern based on koala individual was observed (Fig. 2A; Suppl. Fig. 2B). These finding were further validated by a permutational MANOVA analysis on the unweighted UniFrac distance matrix, which showed that the body region significantly influenced the similarity among our samples (F = 2.7; 10,000 permutations; p = 0.02), while the individual did not (F = 0.75; 10,000 permutations; p = 0.60). Furthermore we estimated the robustness of the UPGMA tree by jackknife and confirmed the clustering of the samples according to body region, with the faecal and the eye samples clustering with maximal jackknife support (100%), while the nodes regarding the rectal and the oral samples being less resolved (Fig. 2B). Thus, the microbiomes from the same body region were more similar across individuals than microbiomes from different body regions of the same koala, which is consistent with human microbiome studies^22^. Replication of this study on a larger number of koalas would be needed to explore variation in microbiome driven by factors other than body location. Nonetheless, as our samples included one male and one female koala, the lack of significant difference between individuals suggests that sex does not strongly influence the koala microbiome.

### Eye microbiome

**Figure 3:**
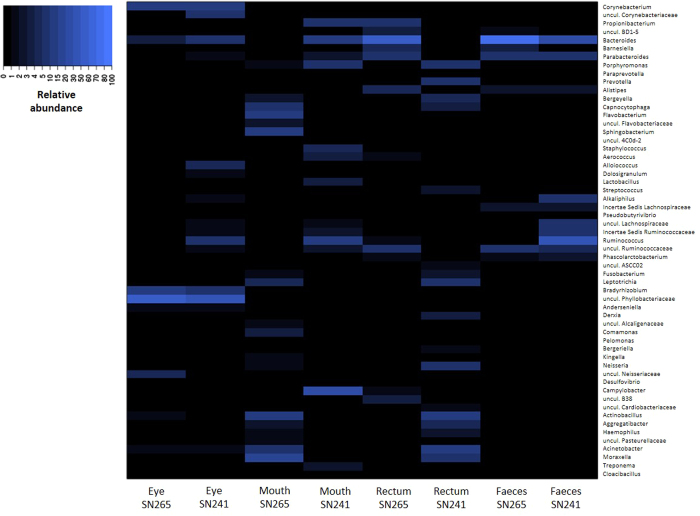
Heatmap analysis of the most abundant bacterial genera detected across all samples. The heatmap depicts the relative percentage of 16S rRNA gene sequences assigned to each bacterial genus (y axis) across the 8 samples analysed (x axis). The heatmap colors represent the relative percentage of the microbial genus assignments within each sample. Square colors shifted towards bright blue indicate higher abundance. The relative abundance values of each genus for each sample are reported in Suppl. Tab. 4.

This is the first study describing the composition of the eye microbiome of a non-human mammal by high-throughput sequencing. We found that the koala eye microbiome was generally similar to that of humans. The eye community had the highest biodiversity among our samples as assessed by the number of OTUs, Phylogenetic Distance (PD) and Shannon index, and low Evenness (Suppl. table 2; Supp Fig. 3; Suppl. table 3). This implies that koala eyes are characterized by a diverse microbial community with a relatively small number of very abundant genera, similar to humans^23^. Furthermore, the community profile at the phylum level was similar between the two koala ocular samples, with representatives of Proteobacteria (76.6 and 51.1%, for SN265 and SN241, respectively) and Actinobacteria (14.9–17.2%) reaching high abundance (Fig. 1; Supp Fig. 1; Suppl. Tab. 1), which is consistent with the few existing human studies^23^,^24^. At the genus level, ocular communities were rich in *Corynebacterium* and *Bradyrhizobium* (Fig. 3; Supp Fig. 4; Suppl. Tab. 4), which were found as common ocular bacteria in humans^23^,^24^. Nevertheless, 35 to 55% of all sequences from the koala eye were represented by uncultured bacteria from the family Phyllobacteriaceae, a group never described before in the eye. In humans it has been reported that the cultivable microbiota of the ocular surface is at a lower proportion than at many other mucosal sites (e.g. the oral cavity) suggesting that ocular communities harbor a hidden microbial diversity^25^. Our findings demonstrated that high-throughput culture-independent analysis of the ocular microbiome has the potential to unravel such diversity. Taking advantage of this methodology, our study sets a baseline for the koala eye microbiome to which microbiomes of diseased states can be compared. Keratoconjunctivitis indeed is one of the main consequences of the highly prevalent *Chlamydia* infection in koalas^19^,^20^ and is likely to be the result of *Chlamydia* interplay with the resident bacteria constituting this complex and diverse microbiota.

### Oral microbiome

In contrast to the eye, the oral microbiome has been well characterized in several other mammalian species and our results show that the composition of the oral microbial community in koalas shares several common features with other mammalian species, including herbivores (wallabies), omnivores (pigs, apes and humans) and carnivores (dogs). Together, Proteobacteria and Bacteroidetes accounted for over 90% of the detected bacteria in SN265 and 56% in SN241, with the remaining belonging mainly to the phylum Firmicutes (31.64%) (Fig. 1; Suppl. Fig. 1; Suppl. Tab. 1). These three phyla were also the main components of the oral microbiome in the majority of other mammalian species. The high abundance of Proteobacteria (30.4–50.9%) that we detected is consistent with previous reports in tammar wallabies^26^, pigs^27^, great apes and humans^16^. Bacteroidetes were also abundant (26.1–40.5%) as found in canine^28^,^29^ and human studies^30^,^31^. Firmicutes were only abundant in SN241 (31.64%), consistently with human, pig, dog and wallaby microbiomes (17.8–52.3%)^26^,^27^,^28^,^30^,^31^,^32^,^33^. The koala oral samples presented low microbial diversity according to alpha diversity measures (Suppl. Fig. 3; Suppl. Tab. 2; Suppl. Tab. 3). In this respect, the data contrasts with the human oral cavity which was found to have the highest OTU richness and phylogenetic diversity within the gastrointestinal tract^34^. At the genus level, qualitative differences were noticeable between the two koala individuals (Fig. 3; Suppl. Fig. 4; Suppl. Tab. 4). However, the main genera present in each individual have been described in other mammalian species. For example, the majority of microbes were members of *Actinobacillus* and *Moraxella* in individual SN265, which are common oral bacteria in wallabies^26^, pigs^27^ and dogs^29^. *Flavobacterium*, which also had high abundance in the same koala (SN265), belongs to the family *Flavobacteriaceae* which includes typical inhabitants of the mammalian oropharyngeal flora (e.g. *Capnocytophaga*)^35^. However, in individual SN241, *Campylobacter*, which is a signature of the human oral microbiota^34^, was the most dominant genus. SN241 exhibited a high abundance of *Porphyromonas*, a resident oral bacteria in dogs^28^ and humans^33^, *Lactobacillus* and *Clostridiales*, also found in pigs and humans^32^. Therefore, the koala oral microbiota does not appear to exhibit unique microbial community structure, despite the diet of Eucalyptus foliage unique to the species.

### Rectal microbiome

The rectal swabs exhibited major differences between the two koalas with SN265 yielding a profile consistent at the phylum level with the few other gut microbiome studies based on the same sample type. The profile of SN265 was dominated by Bacteroidetes (72.0%) and Firmicutes (13.1%), followed by Proteobacteria (6.46%) and Actinobacteria (6.25%) (Fig. 1; Suppl. Fig. 1; Suppl. Tab. 1), similarly to wallabies^36^ and humans^34^,^37^. In contrast, Bacteroidetes (26.6%) and Firmicutes (4.1%) were less common in SN241 than in SN265, while Proteobacteria (56.5%) and Fusobacteria (10.79%) were more common (Fig. 1; Suppl. Fig. 1). The most abundant genera in SN265 were *Bacteroides*, *Parabacteroides* and *Ruminococcaceae* (Fig. 3; Suppl. Fig. 4; Suppl. Tab. 4). *Bacteroides* accounts for approximately 25% of the bacterial population of the human gastrointestinal tract^38^ and has been detected, together with *Parabacteroides* and *Ruminococcus*, in the colon of wild koalas as well^14^. The profile of SN241 was dominated by *Prevotella*, *Porphyromonas*, *Acinetobacter* and *Actinobacillus* (Fig. 3; Suppl. Fig. 4; Suppl. Tab. 4). *Prevotella* has also been associated with the human gut microbiome as the dominant group of type 2 enterotype replacing *Bacteroides* or *Ruminococcus* in some individuals^39^. *Porphyromonas* has been detected in wallaby anal opening^36^, while *Acinetobacter* and *Actinobacillus* are human infectious agents. Despite the individual differences, the rectal microbial profiles of the two koalas overlapped in profiles consistent with those observed in other mammals.

### Faecal microbiome

**Figure 4:**
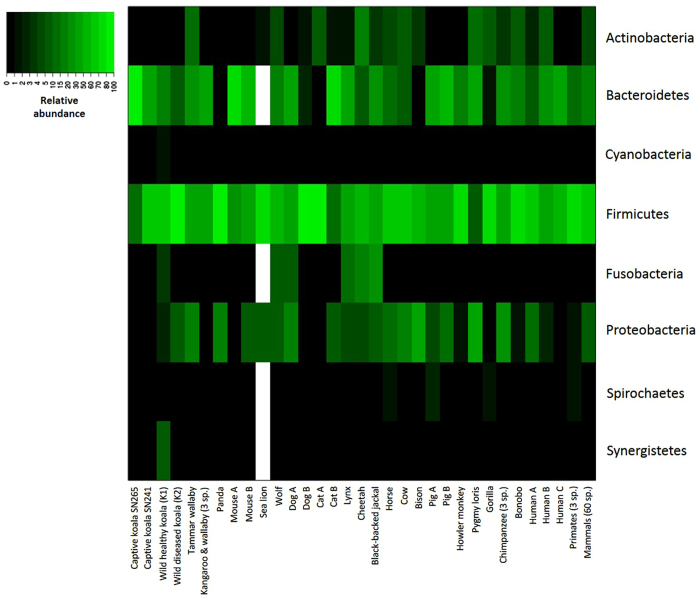
Heatmap analysis of the main bacterial phyla detected across mammalian species. The heatmap depicts the relative percentage of 16S rRNA gene sequences assigned to each bacterial phylum (y axis) across different mammalian species (x axis). The heatmap colors represent the relative percentage of the microbial phylum assignments within each species. Square colors shifted towards bright green indicate higher abundance. When a study reports average abundance values for more species, it is indicated how many species where used in the study. When more than one study per species is available, each study is indicated with a different letter. Blank squares correspond to NA, i.e. not available data, for those phyla for which the abundance values were not reported in the corresponding publication. The relative abundance values of each phylum for each species are reported in Suppl. Tab. 5.

The faecal bacterial communities were dominated by Bacteroidetes (SN265 = 87.6%; SN241 = 34.5%) and Firmicutes (10.9%; 63.6%, respectively) in both koalas (Fig. 1; Suppl. Fig. 1; Suppl. Tab. 1), which is consistent with previously published results on faecal microbiomes of other mammalian species (Fig. 4; Suppl. Tab. 5). A similar composition has been observed in marsupials, including wallabies, kangaroos and also wild koalas^14^,^36^,^40^. Firmicutes are the most predominant phylum in the faecal microbiome of a wide range of mammalian species ranging from 9.4 to 95.4% relative abundance. Bacteriodetes usually occupy the second largest portion of gut microbial communities with abundances varying from 76.2% to 0.6% in bisons. Proteobacteria were detected at very low abundance as previously reported in wild koalas, kangaroos and wallabies, primates, dogs and cats, but in contrast to pandas, pygmy lorises, cows, bisons and chimpanzees, where this phylum represents 15.8 to 30.6% of the total. The relative abundance of Bacteroidetes and Firmicutes varied strongly among the two captive koalas (Fig. 1; Suppl. Fig. 1), but this variation has also been documented within and across other mammalian species (Suppl. Tab. 5). Indeed, the Firmicutes to Bacteroidetes ratio (FB ratio) is generally close to 3:1 in mammals, but can change according to different variables such as host species, diet, age or sample type^9^. Here, individual SN265 presented the lowest FB ratio (FB = 0.12) (Suppl. Tab. 5), but was not very different from cats (FB = 0.17)^41^. In contrast, the FB ratio of individual SN241 (FB = 1.84) was well within the range of FB documented for other species and particularly close to the one found in tammar wallaby (FB = 1.48)^36^.

### Inter-individual differences in microbiomes

The origin of the inter-individual differences in microbiome composition observed in mouth, rectal and faecal samples is unclear, but such differences are also detected in other species^42^. For example, 70% of the phylotypes existing in the human gastrointestinal microbiota have been shown to be subject-specific, with no phylotype being present at an abundance higher than 0.5% in all subjects^43^. This variation may result from competitive exclusion of phylotypes belonging to the same functional group which may select taxa differently depending on the internal environment^44^. Accordingly, human gut microbial communities exhibit a functional redundancy, such that even very different bacterial populations can achieve the same function^39^. Another possibility is an underlying but unobserved gastrointestinal pathology that may have altered the microbiome of one or both koalas although both were clinically healthy at the time of sampling. As the same extraction methods and identical independent triplicate PCR protocols were employed for all samples, the hypothesis of a purely methodological origin for the observed variation is unlikely.

### Are faeces a good proxy for the gastrointestinal microbiome?

Faeces are the most commonly used sample type for gut microbiome investigation in mammals. This is the first study to directly compare the gut microbiome profiles obtained by high-throughput sequencing from faecal and rectal samples. We found that rectal and faecal bacterial communities were phylogenetically distinct based on weighted PCoA (Suppl. Fig. 2B), unweighted PCoA (Fig. 2A) and UPGMA (Fig. 2B). This result is consistent with a human study in which samples from the oral and digestive tract clustered strongly by gastrointestinal site and the multiple colonic samples (including rectal samples) were distinct from stool^34^. Our results contrast with those of Barker *et al.* 2013^14^ where stool samples and colon content from a healthy wild koala were not distinct, but the higher sequencing depth of our study increases the sensitivity of the analysis.

For SN265 the rectal swab and the faeces exhibited similar microbiomes at the phylum level, which were dominated by Bacteroidetes (72 & 87.6%, for the abundance in rectum and faeces respectively) followed in abundance by Firmicutes (10.9 & 13.1%) (Fig. 1). In this individual, the rectal swab and faecal sample were thus correlated at the phylum (Spearman´s correlation test, ρ = 0.66, p = 0.027) and genus (ρ = 0.61, p < 0.0001) level when comparing the relative abundances of the most abundant phyla and genera (see methods for details). However, the microbial profiles exhibited by SN241 strongly differed between rectal and faecal samples: Proteobacteria were highly abundant (56.5%) in the rectal swab, but were almost absent (0.7%) in the faecal sample, which conversely was dominated by Firmicutes (63.6%). Firmicutes represented only a minor component in the rectal swab (4.1%). Bacteroidetes were similarly abundant between the two samples (26.6 & 34.5%) (Fig. 1). Accordingly, the profiles of SN241 showed no significant correlation at the phylum level (ρ = 0.24, p = 0.48) and a negative correlation was present at the genus level (ρ = −0.40, p = 0.001). These results are consistent with a previous study which observed that the composition of the microbiota changes as the gut contents are moved through the colon to the rectum and then excreted, with these changes attributed to differences in substrates, pH and water content^45^.

**Figure 5:**
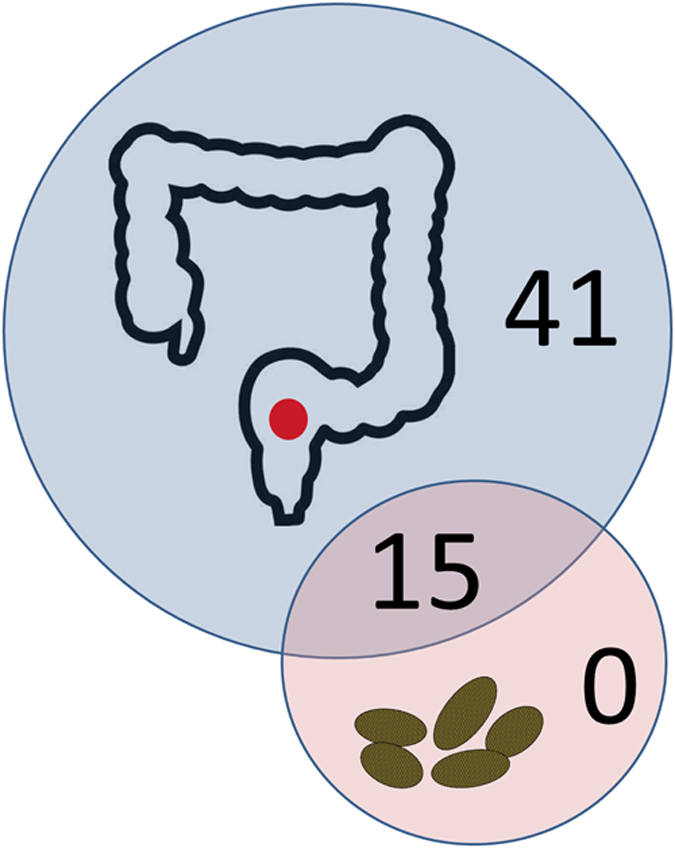
A Venn diagram showing the overlap between the rectal swab and the faeces of koala SN265 in the most abundant genera detected. While 15 genera were shared between rectal swab and faeces, and 41 genera were detected only in the rectal swab, there were no genera unique to the faeces.

The findings here suggest that the microbiota does not simply change, but may lose microbial diversity as it moves from the gut to faeces. Indeed, the rectal swabs had higher diversity than faecal samples according to alpha diversity (Suppl. Fig. 3; Suppl. Tab. 3). Furthermore, when each sample was examined for the presence/absence of the most abundant genera, only 27-34% of the genera that were found in the rectal swabs were also detected in the faecal samples (Suppl. Tab. 6). Accordingly, rectal swabs and faecal samples showed no significant similarity in both koalas when compared both at the phylum and genus level (Jaccard´s index = 0.27–0.6, p > 0.05) (Suppl. Table. 6). A majority (66–73%) of genera found in the rectal swabs were not found in the faeces. Conversely, all the genera that were found in the faeces were found in the rectal swabs, and thus there were no unique genera in the faecal samples (Suppl. Table. 6; Fig. 5). The pattern of presence/absence was exactly the same in both koalas, which was confirmed both at the genus and phylum level (Suppl. Tab. 6) without significant differences between the two koalas (Fisher´s exact test: p = 0.477 for genus level; p ~ 1 for phylum level). Therefore, according to our results, faeces represent only a subsample of the complex bacterial communities inhabiting the gut environment and caution should be used when faecal samples are used to investigate gut microbial diversity.

### Are captive koalas a good proxy for wild microbiome?

The use of captive animals as representative of wild individuals has practical and logistical benefits, particularly for microbiome research where access to animal samples is facilitated in captivity. However, several lines of evidence suggest that different factors associated with captivity may interfere with gut microbiome composition. For example, obesity is known to cause shifts in gut microbiome composition in humans^46^, mice^47^ and is a possible consequence of captivity in zoos, where food is generally of high-quality and easily available, as reported in lemurs^48^. The artificial nature of the zoo environment can cause dietary and behavioural changes, for example in wide-ranging carnivores for which captivity constrains natural activities such as hunting and ranging, obesity can be a consequence.

In general, differences in gut microbiome composition of wild and captive individuals can be expected for species for which diet and activity patterns in captivity are markedly different than in nature. Differences between the microbiome of captive and wild animals are however less likely to happen for herbivorous species^49^, as reported in studies comparing the gut microbiomes of wild and captive pandas^8^ and of domestic and feral goats^17^. Accordingly, our results show that captivity does not appear to strongly influence the koala faecal microbiome at the phylum level. Wild koala gastrointestinal samples exhibited the same dominance of Firmicutes and Bacteroidetes detected in captive koalas with a FB ratio changing considerably across different areas of the hindgut but close to 3:1 in the faeces of the healthy individual^14^. In the diseased wild koala, Firmicutes were even more dominant. At the genus level the profiles of wild and captive koalas were also very similar. In both the present study and Barker *et al.* 2013^14^, Bacteroidetes were mainly represented by *Bacteroides*, *Parabacteroides* and *Alistipes* (Fig. 3; Suppl. Tab. 4), which are common members of the microbiota of mammalian distal intestines. The percentages varied, especially for *Bacteroides*, in accordance with the higher levels of Bacteroidetes detected in one of the captive koalas. The majority of Firmicutes were identified as unknown (*Incertae sedis*) and uncultured Clostridiales. Many Firmicutes in the present study were assigned to the family Lachnospiraceae which is abundant in the digestive tracts of many mammals^50^. Except for the captive koala individual where Firmicutes had low abundances (SN265), *Ruminococcus* dominated. Not surprisingly this genus includes important cellulose-degrading species^51^. *Phascolarctobacterium*, which was originally isolated from koala faeces, but is also broadly distributed in human gastrointestinal tract as subdominant member^52^, was also identified in both this study and Barker *et al.* 2013^14^. Consistently the faecal microbial profiles of the two wild and the two captive koalas were significantly correlated (ρ = 0.64–0.94, p = 0.0001–0.033) at the phylum level (Suppl. Tab. 7). The phyla presence/absence profiles were almost identical in the four different koalas with only 10 differences among the 66 possible pairwise comparisons between the four koalas (Suppl. Tab. 8) showing a very consistent bacterial community composition across the four different samples. Accordingly each pair of samples compared showed high significant similarity (Jaccard´s index = 0.8–1, p < 0.05) (Suppl. Tab. 9). Therefore we can conclude that captivity does not result in major alterations of koala gut microbiome compared to wild conditions when determined from faecal samples.

Our findings therefore suggest that koalas do not face diet related microbiome alterations in captivity. In zoos they are fed a diet almost identical to their natural one, which is based almost exclusively on Eucalyptus leaves. Koalas have evolved an adaptive flexibility that enables them to exploit various Eucalyptus species with a preference of about 50 different varieties out of over 800 existing ones. This diet is easily reproducible in zoos and koalas from this study, for example, were regularly fed 54 different species of Eucalyptus (personal communication of the zoo curator). We conclude that its unique diet, combined with a sedentary lifestyle facilitates koala nutritional management in captivity compared to other mammals and this is reflected in the similarity between wild and captive koala microbiomes.

## Conclusion

The current study compared the microbial communities from multiple body regions of two captive koalas, including the eye and rectum, which are rarely described in the literature of mammal microbiome research. Therefore, getting a wider range of sample types per koala rather than a single sample type from multiple individuals was the priority. The results suggest that the koala eye microbiome is similar to that of other mammals though with some unique aspects observed. The oral and rectal microbiomes do not indicate any major shift in bacterial content that might be attributable to strong adaptive pressure from the Eucalyptus diet. However, the faecal microbiomes represented a subset of rectal microbial diversity suggesting the benefits of non-invasive samples such as faeces may be outweighed by the mixed gastrointestinal compartment origin of faecal bacteria. Nevertheless, captivity did not shift microbiome communities in koalas. Overall, we recommend future microbiome studies in koalas to be based on high-throughput sequencing applied to non-faecal samples. Due to the variation among individuals and the difficulty of obtaining wild koala samples, we suggest that analysis of captive individuals may be more appropriate for clarifying the numerous sources of koala microbial variation.

## Materials and Methods

### Koala Samples

Conjunctival, oral, rectal swabs and faecal samples were obtained from two captive koalas from the Tiergarten Schönbrunn in Vienna, Austria: Bilyarra (Pci-SN241; where “SN” is the studbook number), a 14 year old male and Mirra Li (Pci-SN265), a 12 year old female. The sample size was constrained by the small size of the captive koala population in Europe, by the fact that that most zoos have few koalas in their collections, and by the difficulty of collecting invasive samples, such as rectal and conjunctival swabs. The two koalas were healthy with no pathological condition observed after blood tests, serological, parasitological and bacteriological examination, and had not received any antibiotic treatment for at least the previous 12 years. The faecal samples were collected immediately after defecation. Samples were stored in RNA*later*® solution at room temperature until processed.

### DNA Extraction

Conjunctival, oral and rectal swabs were processed for DNA extraction using a QIAamp® DNA Mini kit (Qiagen, Hilden, Germany) according to manufacturer’s instructions. Genomic DNA was extracted from the faecal samples using a NucleoSpin® Tissue kit (Macherey-Nagel, Düren, Germany) following the protocol provided by the supplier. 50 mg of material were used of each faecal sample. DNA concentration was determined with a NanoDrop® (ND-1000) spectrophotometer (Nanodrop Technologies, Wilmington, DE, USA).

### Polymerase Chain Reaction

Universal primers 27F (5´-AGAGTTTGATCCTGGCTCAG-3´) and 338R (5´-TGCTGCCTCCCGTAGGAGT-3´)^53^ were used for PCR amplification of the V1–V2 hypervariable regions of the bacterial 16S rRNA gene. Each sample was amplified in three replicate reactions to minimize stochastic PCR bias. Each 25 μl PCR reaction contained approximately 200 ng of DNA template, 12.5 μl MyTaq HS Mix (2x), 1.5 μl each primer (10 μM) and sterile distilled water to volume. The amplification conditions were as follows: 4 min of initial denaturation at 94 °C, followed by 16–26 cycles (according to the sample) of denaturation at 94 °C for 15 sec, annealing at 55 °C for 20 sec, and extension at 72 °C for 10 sec, with the last cycle followed by a 10 sec extension step at 72 °C. Water was used in the place of a DNA template as a negative control. After being visualized on a 1.5% agarose gel, the three replicate PCR products for each sample were pooled and purified using the MSB® Spin PCRapace kit (STRATEC Molecular GmbH, Berlin, Germany) according to manufacturer’s protocol and eluted in 25 μl elution buffer. The PCR negative samples were also pooled and treated as a sample to monitor possible PCR contamination.

### Illumina Library Preparation and Sequencing

Illumina sequencing libraries were generated as described in Meyer and Kircher 2010^54^ with some modifications as described in Supplementary Methods. The libraries were first amplified in a 50 μl volume reaction using 5 μl of DNA library, 0.5 μl Herculase II Fusion DNA Polymerase (Agilent Technologies Inc.), 10 μl Herculase II Reaction Buffer (5x), 0.5 μl dNTPs (25 mM), 1 μl Single Index Primer P5 (10 μM), 1 μl Illumina Index Primer P7 (10 μM) and sterile distilled water to volume. A unique P7 Index Primer was used for each library to allow for subsequent sample discrimination after the sequencing of pooled libraries. Each library was amplified in three replicate reactions to minimize amplification bias in individual PCRs. PCR cycling conditions consisted of initial denaturation for 5 min at 95 °C, followed by 10 cycles of 30 sec denaturation at 95 °C, 30 sec annealing at 60 °C and 40 sec elongation at 72 °C. A final 7 min elongation step at 72 °C completed the reaction. The three replicate PCR products for each sample were pooled and purified using the QIAquick PCR Purification Kit (Qiagen, Hilden, Germany) and eluted in 40 μl elution buffer. A negative control extraction library was also prepared and indexed separately to monitor any contamination introduced during the experiment.

Amplified libraries were quantified using the 2200 TapeStation (Agilent Technologies Inc.) on D1K ScreenTapes. The indexed DNA libraries were then pooled at equimolar concentrations for paired-end sequencing (2 × 250) on an Illumina MiSeq v2 platform at the Danish National High-Throughput DNA Sequencing Centre in Copenhagen, Denmark.

### Ethics Statement

All experiments involving koala tissues were approved by the Internal Ethics Committee of the Leibniz Institute for Zoo and Wildlife Research, approval number 2012-09-06. All koala samples were obtained in accordance with the approved guidelines of the Leibniz Institute for Zoo and Wildlife Research and of Tiergarten Schönbrunn.

### Bioinformatics and Statistical Analysis

A total of 2,584,237 paired-end sequence reads 250 bp long were generated (Suppl. Tab. 10) and then sorted by index sequences. 87.5% of paired-end reads were successfully merged reads into single reads. After primer and quality trimming, overall 2,064,872 sequences (91.2%) were retained. Quality trimmed reads were analysed using the Quantitative Insights Into Microbial Ecology (QIIME) pipeline software (version 1.6.0) ( http://qiime.org). A further quality filtering step was performed using split_libraries_fastq.py command within the QIIME package to remove reads containing ambiguous bases.

Sequences were clustered into Operational Taxomonic Units (OTUs) based on 97% sequence similarity and the most abundant sequence within an OTU was chosen as the OTU’s representative sequence. The representative sequences were then aligned and taxonomically classified against the SILVA reference database, release108 (SILVA 108; http://www.arb-silva.de). Chimeras were removed from the representative set, together with singletons and chloroplast sequences. Removal of these sequences left a total of 7,843 OTUs (Suppl. Tab. 10). OTUs with an abundance <0.1% of the total read count were removed from the OTU table to simplify the visualization of the results. This way lists of the “most abundant” phyla and genera were generated. Taxonomy summaries with relative abundance data at the phylum and genus level were subsequently generated. More details about bioinformatic software and parameters used are available in the Supplementary Methods.

We calculated alpha and beta diversity metrics along with rarefaction plots from the complete OTU table using QIIME. The rarefaction curves tended to level off after approximately 100,000 reads demonstrating high coverage depth (Suppl. Fig. 5). Alpha diversity indices (within sample diversity) - Phylogenetic Distance, Shannon diversity index and Evenness - were calculated at a sequence depth of 161,378 reads/sample for 10,000 times and then averaged. The selected maximum sampling depth corresponded to the minimum number of quality reads obtained from any individual sample in the dataset. Phylogenetic distance (PD) is a measure of biodiversity that considers phylogenetic difference between species. Evenness measure how equally a community is numerically distributed among the species. Shannon diversity index (H) takes into account both abundance and evenness of species present in a community. Beta diversity (between samples diversity) was estimated by computing from the phylogenetic tree the unweighted and weighted UniFrac distances^55^ between samples at the same sequence depth. UniFrac distances describe the dissimilarity among samples by assessing the evolutionary distances of bacterial phylotypes observed. Unweighted UniFrac only considers the presence/absence of taxa, while weighted UniFrac takes into account the differences in taxa abundance. UniFrac distance matrices were visualised using principal coordinates analysis (PCoA). The Unweighted Pair Group Method with Arithmetic Mean (UPGMA) for clustering of samples was performed as an alternative hierarchical clustering method to interpret the Unifrac distance matrix. The robustness of the UPGMA tree was estimated using jackknife based on 1,000 replicates running the jackknifed_beta_diversity.py workflow in QIIME.

The results from QIIME were further analysed using the statistical software R version 3.1.0 ( http://www.R-project.org). To test whether the body region or the individual had a significant influence on the similarity among our samples measured by unweighted Unifrac distance, we performed a permutational MANOVA using the function adonis of the package “vegan”. We decided to focus only on the unweighted UniFrac because we considered it a safer measurement of similarity. Indeed, this distance is independent of abundance data and therefore less susceptible to variation due to methodology (e.g. PCR). Heatmaps were generated using the heatmap.2 function from the package “gplots”. We calculated the mean and the standard deviation of the three alpha diversity metrics over the 10,000 iterations for each sample. We also measured the mean of the differences of the indices values among the four sample types for each koala across the 10,000 iterations and the 95% confidence intervals of the differences. To assess the similarity between rectal and faecal communities for each koala, the relative abundances of the most abundant phyla and genera detected in each sample were compared. For the comparison between the faecal communities of the captive koalas and of the wild koalas from Barker *et al.* 2013^14^, the relative abundances of the eleven phyla detected in both studies (calculated from complete OTU lists) were used. The comparisons were performed using the Spearman’s rank correlation coefficients (ρ) using the cor.test function in R. Unequal sampling depth could bias the correlation value because of the presence of many low abundance taxa in the samples. Indeed, those taxa may have been detected in samples characterized by higher number of reads set but not in those with less reads. We therefore subsampled (randomly, without replacement) the data sets to match the minimum number of reads in one of the samples so that each sample was represented by the same number of reads. Contingency tables with presence/absence data of the most abundant phyla and genera from the rectal swabs and faecal samples, and the phyla detected in the captive and wild koalas were created in R. Fisher´s exact test was performed in R in order to test if there was any significant difference between the two koalas and between phylum and genus level in the pattern of the distribution of the taxa among rectal swabs and faecal samples. We decided to compare only the phyla detected in our study with the ones detected in Barker *et al.* 2013^14^, but not the genera because the different methods (extraction, PCR primers, sequencing) used in the two studies may not allow a comparison at such fine taxonomic resolution. Jaccard’s coefficient^56^ was also calculated to measure the similarity between the bacterial communities for each pair of samples compared. Jaccard’s index was chosen since we decided not to count *double-zeros*, i.e. the absence of a taxon from two samples, to compute similarity. It ranges from 0 to 1, where 0 means no similarity between the two analysed samples and 1 complete similarity. To determine if the values for the index differed from what would be expected at random, we compared the observed similarity values with the table of statistical significance at P = 0.05 of lower and upper critical values^57^, for the total number of taxa present in either of the two samples being compared.

## Author Contributions

N.A. and A.D.G. designed the project; H.V. collected and provided the samples; N.A. performed all laboratory experiments; N.A. and A.C analyzed the data; N.A., P.T., A.L.R. and A.D.G. discussed the results and wrote the manuscript.

## Additional Information

**How to cite this article**: Alfano, N. *et al.* Variation in koala microbiomes within and between individuals: effect of body region and captivity status. *Sci. Rep.* **5**, 10189; doi: 10.1038/srep10189 (2015).

## Supplementary Material

Supplementary Information
