# Supplementary material for: Variation in koala microbiomes within and between individuals: effect of body region and captivity status
Source: Sci Rep. 2015 May 11;5:10189. doi: 10.1038/srep10189 (PMC4426690; doi:10.1038/srep10189)
Supplement: Supplementary Information [file srep10189-s1.doc]

**Variation in koala microbiomes within and between individuals: effect of body region and captivity status.**

Niccoló Alfano^1^, Alexandre Courtiol^1^, Hanna Vielgrader^2^, Peter Timms^3^, Alfred L. Roca^4^, Alex D. Greenwood^1^*

^1^ Leibniz Institute for Zoo and Wildlife Research, Berlin, Germany

^2^ Tiergarten Schönbrunn, Vienna, Austria

^3^ University of the Sunshine Coast, Sippy Downs, Queensland, Australia

^4^ Department of Animal Sciences, University of Illinois at Urbana-Champaign, Urbana, Illinois, USA

* Correspondence to: Alex D. Greenwood, Department of Wildlife Diseases, Leibniz Institute for Zoo and Wildlife Research, Alfred-Kowalke-Str. 17, 10315 Berlin, Germany, +49 (0)30 5168255, [greenwood@izw-berlin.de](mailto:greenwood@izw-berlin.de)

**Supplementary Methods**

**Illumina Library Preparation**

Illumina sequencing libraries were generated as described in Meyer and Kircher 2010 with the following modifications. Blunt ending reactions were set up in a 50 μl volume containing 1 μg of pooled purified PCR product, 6 μl NEBuffer 2 (10x), 0.6 μl dNTPs (25mM), 7 μl ATP (10mM), 5 μl BSA (10mg/ml), 3 μl T4 Polynucleotide Kinase (10U/μl), 2 μl T4 DNA Polymerase (3U/μl) and sterile distilled water to volume. The reaction was incubated at 25°C for 15 min, followed by 15 min at 12°C. The blunt-ended DNA was then purified using the MinElute PCR Purification Kit (Qiagen, Hilden, Germany) and eluted in 20 μl elution buffer. Blunt-ended DNA was then ligated to Illumina multiplex adapters (Illumina Inc.)(5´-ACA CTC TTT CCC TAC ACG ACG CTC TTC CGA TCT and 5´-AGA TCG GAA GAG C for one adapter, 5´-GTG ACT GGA GTT CAG ACG TGT GCT CTT CCG ATC T and 5´-AGA TCG GAA GAG C for the other) in a 40 μl reaction containing 20 μl Quick Ligase Buffer (2x), 1 μl Quick Ligase (5U/μl) and 1 μl Illumina adapter mix (10 μM). The reaction was incubated at room temperature for 20 min. The adapter-ligated DNA was then purified using the MinElute PCR Purification Kit (Qiagen, Hilden, Germany) and eluted in 35 μl elution buffer. Finally the blunt-ended adapter-ligated DNA went through a 40 μl adapter fill-in reaction containing 4 μl Thermopol Buffer (10x), 1 μl dNTPs (25 mM) and 2 μl Bst Polymerase (8U/μl). The reaction was incubated at 65°C for 20 min, followed by 20 min at 80°C. All reagents used in library preparation were from New Englands Biolabs® Inc., Ipswich, MA, USA.

**Bioinformatics**

Paired-end reads were merged reads into single reads using the FLASH software tool (http://ccb.jhu.edu/software/FLASH/) using 10 bp as minimum overlap and 0.1 as maximum allowed ratio between the number of mismatched base pairs and the overlap length. Primers were removed from the reads using Cutadapt (https://code.google.com/p/cutadapt/): reads that did not contain the primers or with more than a mismatch in the primer sequence were discarded and reads between 250–370 bp in length were retained. Low quality bases were trimmed using FASTX-Toolkit (FASTQ Quality Filter tool) (http://hannonlab.cshl.edu/fastx_toolkit/): only reads with at least 75% of read length with quality score above 30 were kept.

Within QIIME package, sequences were clustered using UCLUST into Operational Taxomonic Units (OTUs) based on 97% sequence similarity through open-reference OTU picking against the Greengenes database (version 12_10) (http://greengenes.lbl.gov/). The most abundant sequence within an OTU was chosen as the OTU’s representative sequence. The representative sequences were then aligned against the 16S rRNA Greengenes core set using PyNAST with a minimum identity of 75%. Representative sequences were taxonomically classified using BLAST against the SILVA reference database, release108 (SILVA 108; http://www.arbsilva.de). The alignment was then filtered to remove gaps and a maximum-likelihood phylogenetic tree was constructed from the filtered alignment using FastTree. Chimeras were removed from the representative set using Chimera Slayer.

**Supplementary Figures**

**Supplemental Figure 1**


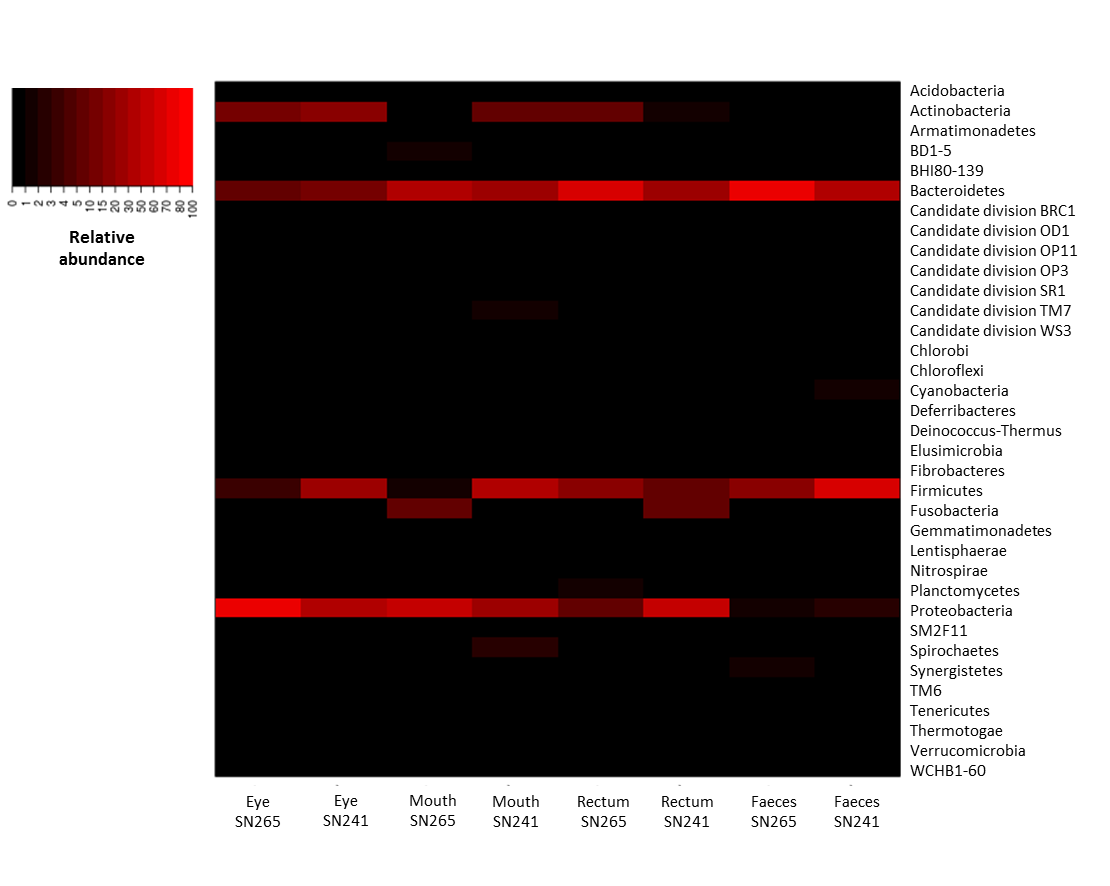


**Supplemental Figure 1.** **Heatmap analysis of the complete list of bacterial phyla detected across all samples**. The heatmap depicts the relative percentage of 16S rRNA gene sequences assigned to each bacterial phylum (y axis) across the 8 samples analysed (x axis). The heatmap colors represent the relative percentage of the microbial phylum assignments within each sample. Square colors shifted towards bright red indicate higher abundance.

**Supplemental Figure 2**


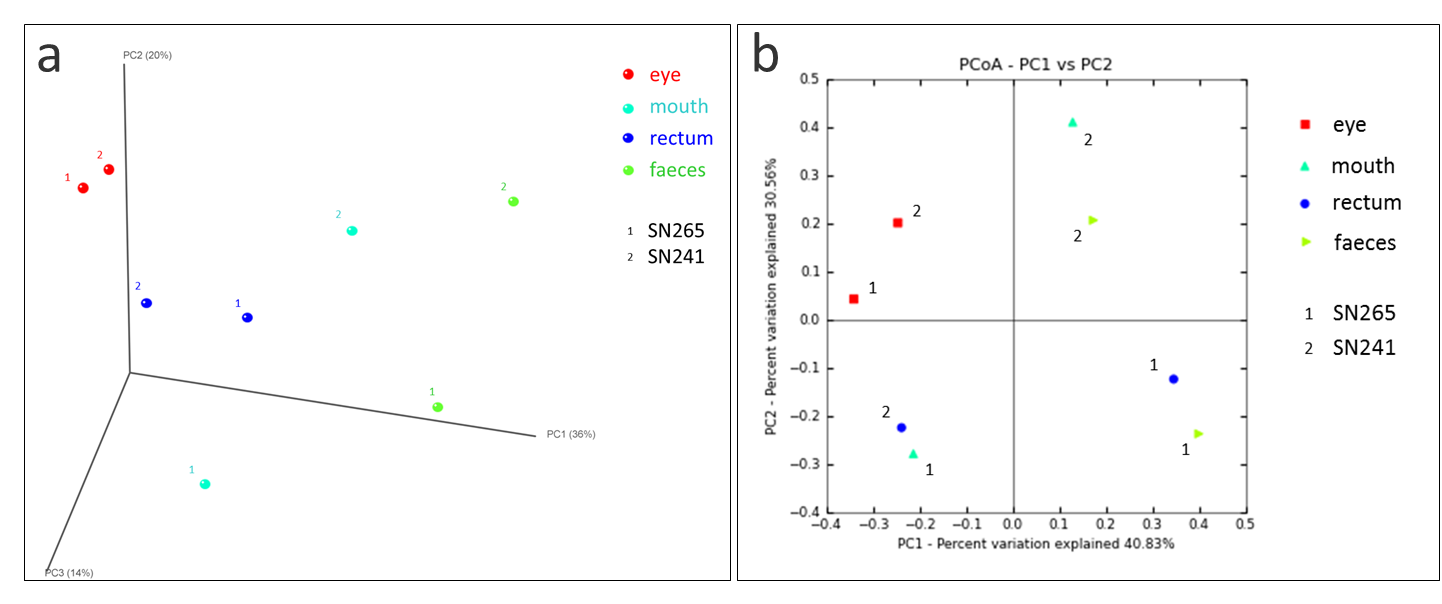


**Supplemental Figure 2.** **Principal Coordinate Analysis (PCoA) of Unifrac distances of the eye, mouth, rectal and faecal samples of two captive koalas**. Panel A is a 3 dimensional unweighted PCoA plot which is plotted by sample type. Panel B shows the 2 dimensional weighted PCoA plot by sample type.

**Supplemental Figure 3**

**
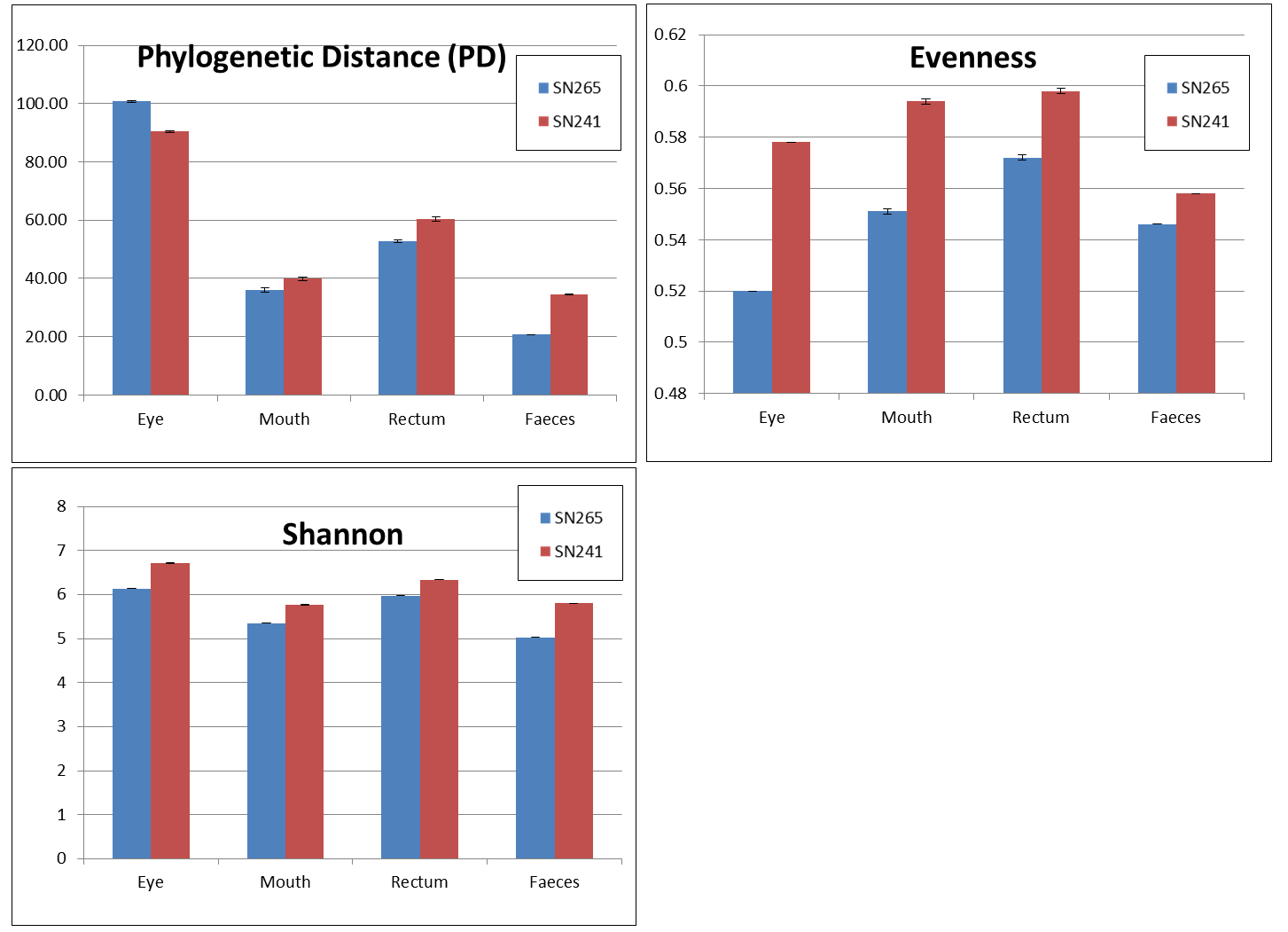
**

**Supplemental Figure 3.** **Measures of alpha diversity of the eye, mouth, rectal and faecal bacterial communities of the two koalas**. Phylogenetic distance (PD), Shannon diversity index and Evenness are shown in the respective panels. Each alpha diversity metric is presented as the mean value of the 10,000 iterations at the rarefaction depth of 161,378 sequences/sample. The error bars represent the standard deviations.

**Supplemental Figure 4**


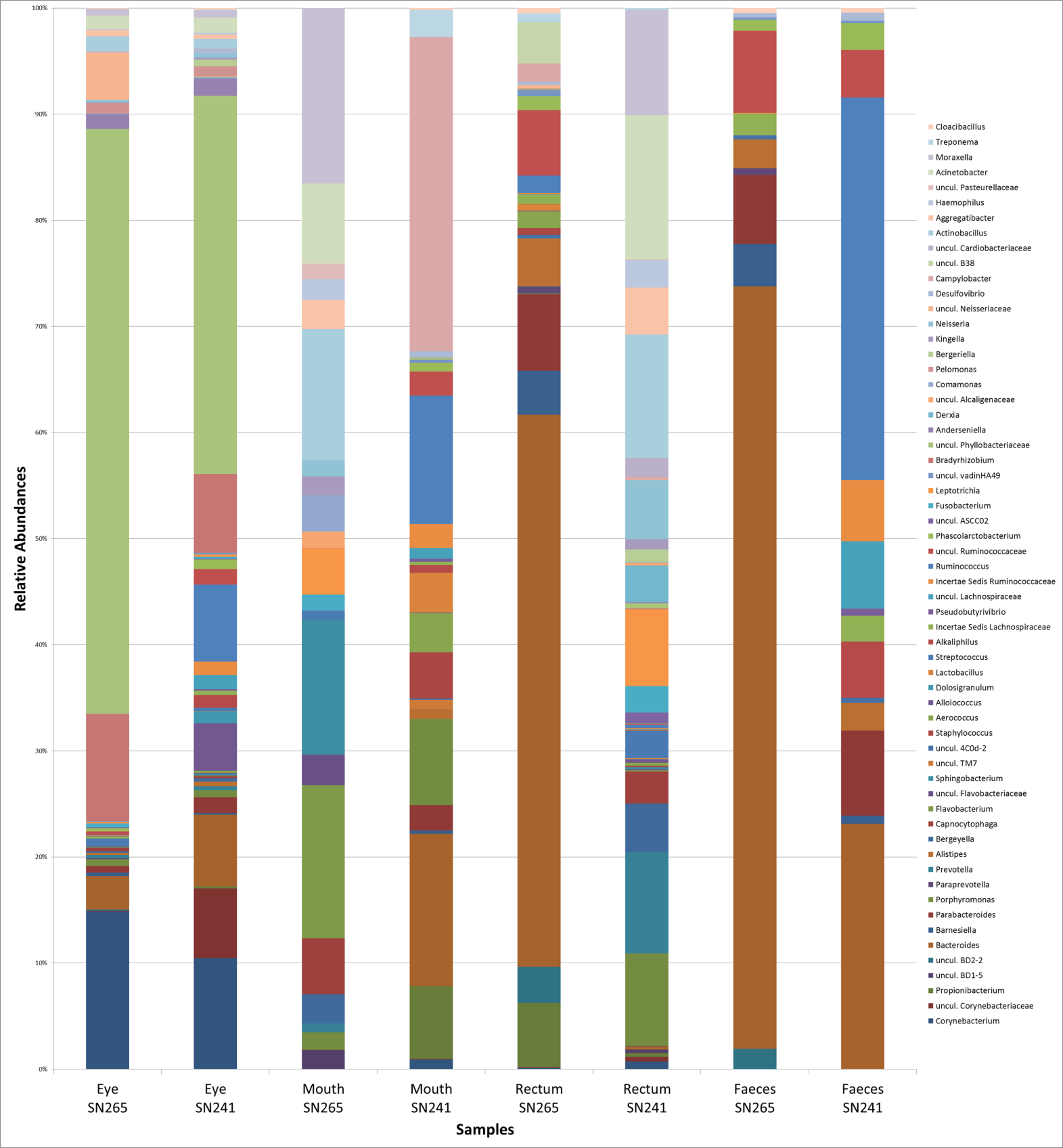


**Supplemental Figure 4.** **The most abundant bacterial genera found in each sample**. Bar chart representations are shown of the relative abundances of the most abundant bacterial genera found in the eye, mouth, rectum and faeces of the two koalas in the present study.

**Supplemental Figure 5
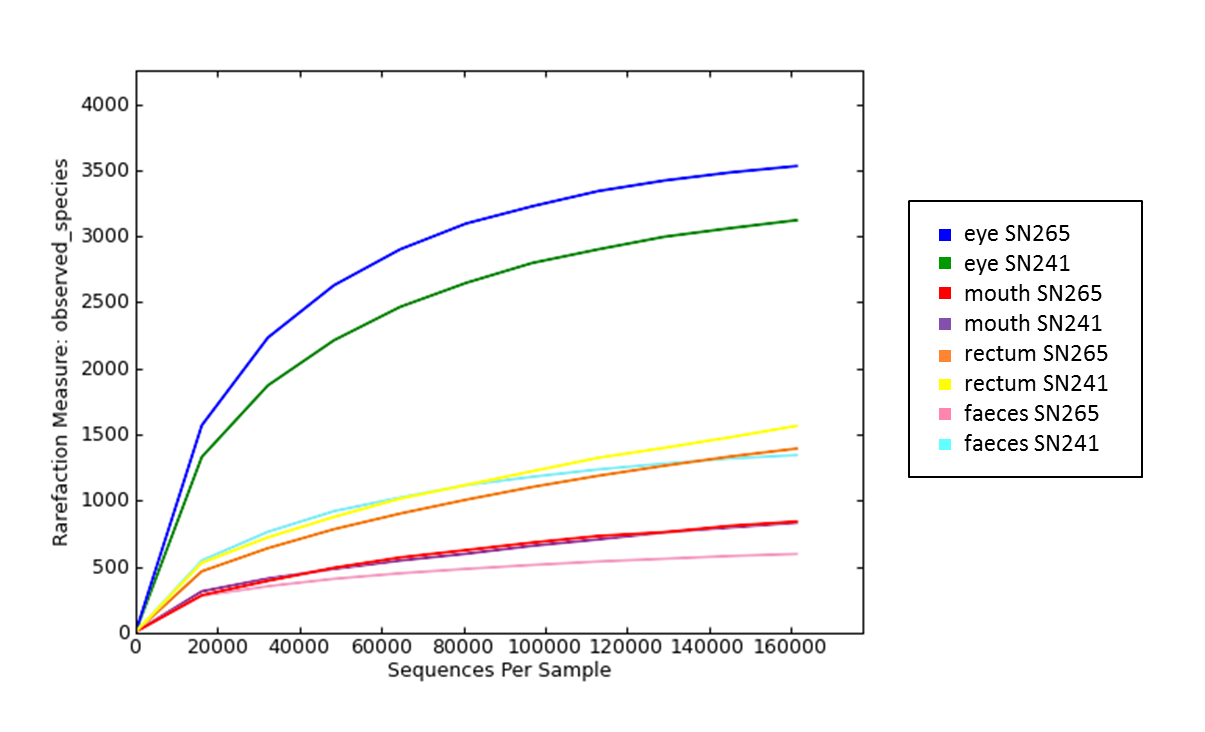
Supplemental Figure 5.** **Rarefaction analysis of the different samples sequenced.** Rarefaction curves obtained from the eye, mouth, rectum and faeces of the two koalas showing the number of unique OTUs (observed species metric) as a function of sequencing depth for each sample. The curves were calculated at a maximum rarefaction depth of 161,378 sequences/sample.

**Supplementary Tables**

**Suppl. Tab 1 Relative abundance of most abundant OTUs at phylum level in the eye, mouth, rectal and faecal microbiome from the two koalas.**

Data derived from OTU table where OTUs with an abundance <0.1% of the total read count were removed in order to semplify the visualization of the results.

**Suppl. Tab. 2 Numbers of OTUs and measures of alpha diversity of the eye, mouth, rectal and faecal bacterial communities of the two koalas.**

Each alpha diversity metric - Phylogenetic distance, Shannon diversity index and Evenness - is presented as the mean value of the 10,000 iterations at the rarefaction depth of 161,378 sequences/sample.

**Suppl. Tab. 3 The differences in each alpha diversity metric between the eye, mouth, rectal and faecal samples of each koala.**

The mean of the differences of the indices values (across the 10,000 iterations) among the eye, mouth, rectal and faecal bacterial communities of the two koalas for each of the three alpha diversity metrics measured - Phylogenetic distance (PD), Shannon diversity index and Evenness measure. Values above the diagonal concern individual SN265, while those below SN241. The 95% confidence intervals of the differences is given as well.

**Suppl. Tab. 4 Relative abundance of most abundant OTUs at genus level in the eye, mouth, rectal and faecal microbiome from the two koalas.**

Data derived from OTU table where OTUs with an abundance <0.1% of the total read count were removed in order to simplify the visualization of the results.

**Suppl. Tab. 5 Relative abundances of the main bacterial phyla detected in the faecal microbiomes of several mammalian species.**

For each taxon, the number of individuals examined, the sequencing method and the sample type used are indicated. In those studies where sequences were split between Bacteroidetes/Chlorobi and Bacteroidetes groups, those data were pooled into Bacteroidetes. "NA" is used for not available abundance data. References: wild koalas^1^; tammar wallaby^2^; kangaroo and wallaby^3^; panda^4^; mouse A^5^; mouse B^6^; sea lion^7^; wolf^8^; dog A^9^; dog B^10^; cat A^10^; cat B^11^ ; lynx^12^; cheetah^13^; black-backed jackal^13^; horse^14^; cow^15^; bison^16^; pig A^17^; pig B^18^; howler monkey^19^; pigmy loris^20^; gorilla^21^; chimpanzee^22^; bonobo^22^; human A^22^; human B^23^; human C^24^; primates^25^; mammals^26^.

**Suppl. Tab. 6 Frequency distribution of the most abundant genera and phyla in the rectal swabs and faeces of the two koalas.**

Contingency tables showing the frequency distribution of the binary variables defined as the presence/absence of SN265´s most abundant bacterial genera (a) and phyla (c), and of SN241´s most abundant genera (b) and phyla (d) from the rectal and faecal samples. Below the tables is indicated the p-value of the Fisher´s exact test performed to test if there was any significant difference between the contingency tables of the two koalas both at genus and phylum level. Jaccard Index computed from table a = 0.27 (N=60; C.I. 95%: 0.21-0.46); from table b = 0.34 (N=60; C.I. 95%: 0.22-0.45); from table c = 0.6 (N=11; C.I. 95%: 0-0.7); from table d = 0.54 (N=11; C.I. 95%: 0-0.64).

**Suppl. Tab. 7 Correlation between the faecal samples of the two captive and two wild koalas.**

Correlation matrix of the relative abundances of the eleven phyla detected both in the faecal samples of the two captive koalas from this study (SN265 and SN241) and the two wild koalas from Barker et al. 2013 (K1 and K2). Correlation was measured using the Spearman's rank correlation coefficients. The p-values are given in brackets. The significant correlations (p ≤ 0.05) are presented in bold characters.

**Suppl. Tab. 8 Phyla distribution in the faecal samples of the two captive and two wild koalas.**

Contingency tables showing the presence/absence distribution of the eleven phyla detected both in the faeces of the two captive koalas analysed in this study (SN265 and SN241) and of the two wild koalas from Barker et al. 2013 (K1 and K2).

**Suppl. Tab. 9 Similarity between the faecal samples of the two captive and the two wild koalas.**

Jaccard´s coefficient of similarity between bacterial presence/absence profiles between faecal samples of captive (SN265 and SN241 from this study) and wild koalas (K1 and K2, from Barker et al. 2013). The table shows the lower and upper critical values of the coefficient with a probability level of P < 0.05 considering the total number of taxa present in either of the two samples being compared (N). * Significant values.

**Suppl. Tab. 10 Statistics of the raw and quality filtered sequences from Illumina sequencing of the eye, mouth, rectal and faecal samples of the two koalas.**

**Supplementary References**

1. Barker, C. J., Gillett, A., Polkinghorne, A. & Timms, P. Investigation of the koala (*Phascolarctos cinereus*) hindgut microbiome via 16S pyrosequencing. *Vet. Microbiol.* **167**, 554-64 (2013).

2. Chhour, K. L., Hinds, L. A., Deane, E. M. & Jacques, N.A. The microbiome of the cloacal openings of the urogenital and anal tracts of the tammar wallaby, *Macropus eugenii*. *Microbiol.* **154**, 1535-43 (2008).

3. Gulino, L. M. *et al.* Shedding light on the microbial community of the macropod foregut using 454-amplicon pyrosequencing. *PLoS One* **8**, e61463 (2013).

4. Zhu, L., Wu, Q., Dai, J., Zhang, S. & Wei, F. Evidence of cellulose metabolism by the giant panda gut microbiome. *Proc. Natl. Acad. Sci. U. S. A.* **108**, 17714-9 (2011).

5. Schloss, P. D. *et al.* Stabilization of the murine gut microbiome following weaning. *Gut Microbes* **3**, 383-93 (2012).

6. Linnenbrink, M. *et al.* The role of biogeography in shaping diversity of the intestinal microbiota in house mice. *Mol. Ecol.* **22**, 1904-16 (2013).

7. Lavery, T. J., Roudnew, B., Seymour, J., Mitchell, J. G. & Jeffries, T. High nutrient transport and cycling potential revealed in the microbial metagenome of Australian sea lion (*Neophoca cinerea*) faeces. *PLoS One* **7**, e36478 (2012).

8. Zhang, H. & Chen, L. Phylogenetic analysis of 16S rRNA gene sequences reveals distal gut bacterial diversity in wild wolves (*Canis lupus*). *Mol. Biol. Rep.* **37**, 4013-22 (2010).

9. Swanson, K. S. *et al.* Phylogenetic and gene-centric metagenomics of the canine intestinal microbiome reveals similarities with humans and mice. *Isme J.* **5**, 639-49 (2011).

10. Handl, S., Dowd, S. E., Garcia-Mazcorro, J. F., Steiner, J. M. & Suchodolski, J. S. Massive parallel 16S rRNA gene pyrosequencing reveals highly diverse fecal bacterial and fungal communities in healthy dogs and cats. *FEMS Microbiol. Ecol.* **76**, 301-10 (2011).

11. Tun, H. M. *et al.* Gene-centric metagenomics analysis of feline intestinal microbiome using 454 junior pyrosequencing. *J. Microbiol. Methods* **88**, 369-76 (2012).

12. Alcaide, M. *et al.* Gene sets for utilization of primary and secondary nutrition supplies in the distal gut of endangered Iberian lynx. *PLoS One* **7**, e51521 (2012).

13. Menke, S. *et al.* Oligotyping reveals differences between gut microbiomes of free-ranging sympatric Namibian carnivores (*Acinonyx jubatus*, *Canis mesomelas*) on a bacterial species-like level. *Front. Microbiol.* **5**, 526 (2014).

14. Costa, M. C. *et al.* Comparison of the fecal microbiota of healthy horses and horses with colitis by high throughput sequencing of the V3-V5 region of the 16S rRNA gene. *PLoS One* **7**, e41484 (2012).

15. Mao, S., Zhang, R., Wang, D. & Zhu, W. The diversity of the fecal bacterial community and its relationship with the concentration of volatile fatty acids in the feces during subacute rumen acidosis in dairy cows. *BMC Vet. Res.* **8**, 237 (2012).

16. Weese, J. S., Shury, T. & Jelinski, M. D. The fecal microbiota of semi-free-ranging wood bison (*Bison bison athabascae*). *BMC Vet. Res.* **10**, 120 (2014).

17. Lamendella, R., Oerther, D. B., Martinson, J., and Santo Domingo, J. W. Comparative fecal metagenomics reveals previously unknown functionality of the distal swine gut. *BMC Microbiol.* **11**, 103 (2011).

18. Looft, T. *et al.* In-feed antibiotic effects on the swine intestinal microbiome. *Proc. Natl. Acad. Sci. U. S. A.* **109**, 1691-6 (2012).

19. Amato, K. R. *et al.* Habitat degradation impacts black howler monkey (*Alouatta pigra*) gastrointestinal microbiomes. *Isme J.* **7**, 1344-53 (2013).

20. Xu, B. *et al.* Metagenomic analysis of the pygmy loris fecal microbiome reveals unique functional capacity related to metabolism of aromatic compounds. *PLoS One* **8**, e56565 (2013).

21. Frey, J. C. *et al.* Fecal bacterial diversity in a wild gorilla. *Appl. Environ. Microbiol.* **72**, 3788-92 (2006).

22. Ochman, H. *et al.* Evolutionary relationships of wild hominids recapitulated by gut microbial communities. *PLoS Biol* **8**, e1000546 (2010).

23. Arumugam, M. *et al.* Enterotypes of the human gut microbiome. *Nature* **473**, 174-80 (2011).

24. Eckburg, P. B. *et al.* Diversity of the human intestinal microbial flora. *Science* **308**, 1635-8 (2005).

25. Yildirim, S. *et al.* Characterization of the fecal microbiome from non-human wild primates reveals species specific microbial communities. *PLoS One* **5**, e13963 (2010).

26. Ley, R. E. *et al.* Evolution of mammals and their gut microbes. *Science* **320**, 1647-51 (2008).
